# Case of Spinal Irritation

**Published:** 1873-01

**Authors:** S. Weed

**Affiliations:** Clyde; Wayne County, N. Y.


BUFFALO

### Medical and Surgical Journal.

VOL XII. JANUARY, 1873. No. 6

### Original Communications.

:o:

ART. I.—*Case of Spinal Irritation.* S. WEED, M. D., Wayne County, N. Y.

Read before the Central New York Medical Association.

It is generally conceded, I believe, that the treatment of what the books call "Spinal Irritation" is not infrequently quite unsatisfactory. Hence my apology for reporting the subjoined case is its happy termination, and also to bring to the notice of this society a therapeutic agent which, perhaps, in well selected cases, may be found worthy of trial in some of the phases of this protean malady.

I first saw Mrs. C., a married lady of twenty-nine years, as an invalid, at her parents', in Clyde, September 7th, 1872. I was hurriedly called in while passing to prescribe for her temporarily, she being under the care of another physician and he out of town. I found her hands, feet and stomach in a sort of tonic spasm. Her sufferings were great. I left two powders of sulphate of morphine of about one-third grain each, with directions that one should be taken immediately and the other in three or four hours if complete relief should not be obtained from the first. I then left without further investigating the case.

SEPT. 11TH.—Was requested to visit and prescribe for Mrs. C. again, as her physician was again out of town. On arriving at the bedside I learned that she was suffering from a somewhat different train of symptoms from those most prominent when I had before seen her. The pain or distress under which she was now laboring appeared to be confined mostly to the region of the stomach and chest. Body apparently well nourished; surface of nearly natural temperature; face somewhat flushed; pulse 65 per minute; tongue coated with a whitish fur; loss of appetite; nights passed without sleep unless opiates were taken, and had been confined to the bed since my former hurried call. She stated that she had been an invalid from January last, when she thought that she had overstrained her back in lifting a sick sister. She had been treated or prescribed for by five or six doctors during that time.

On firm pressure I found much tenderness of the spine, extending from the lower cervical to the lower dorsal vertebra, and producing great distress in the stomach, chest and arms. Repeated prescription of former call, as I was requested to relieve the symptoms only then present and most urgent.

SEPT. 14TH.—Was requested this day to take full charge of the case. Mrs. C. had been confined strictly to the bed since my last visit. Had had no rest except while under the influence of morphine.

Ordered an ice-bag to be prepared from a beef's bladder and placed in the leg of a woolen stocking, filled with pounded ice and applied to the tender spine twice a day, night and morning, and from one to two hours at a time. Chapman's spinal rubber ice-bag would have been more convenient and preferable, but this I could not obtain.

SEPT. 15TH.—On calling this morning, found patient with ice-bag in position. Had had it in position for about an hour. Various articles had been placed on either side of it to prevent undue pressure. She stated that the sensation produced was that of a dull ache.

SEPT. 16TH.—Patient sitting up, or rather partially reclining on the sofa, and feeling every way better. To continue the treatment as before directed until seen again.

SEPT. 18TH.—Patient decidedly better. Sleeps well and has a good appetite.

SEPT. 19TH.—Rode home in a one-horse carriage—distance 5 miles.

SEPT. 22D.—Called in passing and found Mrs. C. still improving. Had not used the spinal ice-bag for a couple of days owing to a difficulty in getting the material to make a new one. The old one had become too offensive for use. Was to use one again that day.

SEPT. 25TH.—Was requested to call this day, as patient was having an ague chill. (I was attending a fever patient near by.) Had been feeling well until taken by the chill. Left quinine and dovers powder.

SEPT. 28TH.—Patient up about the house, and says that she feels perfectly well. That the spinal tenderness and its accompanying sympathetic symptoms are all gone. Had on the spinal ice-bag while attending to her household duties. Directed her to continue its use for sometime to come, at least once a day. Neither anodyne or opiates had been taken after her commencement with the ice.

DEC. 6TH.—Saw Mrs. C. at her parents' to-day. Says that she continued the use of the spinal ice-bag about a week after my last call on her in September. That she has enjoyed almost perfect health from that time to the present. She is now the picture of health. On firm pressure there was, however, still elicited slight tenderness in the lower cervical and two or three of the dorsal vertebra. Her mother assured me that there was no curvature of the spine.

In reporting the above case I have purposely avoided any speculation upon its pathology or treatment, since it is still a mooted question among the ablest in our profession as to whether there is any such disease as spinal irritation.

Dr. John Chapman, of London, was the first to introduce and use the spinal ice-bag in the treatment of a variety of diseases having their origin in the spine or other great nerve centers. In Braithwaite's Retrospect, No. 57, and page 39, are reported a series of cases of neuralgia cured by the use of the spinal bag—mostly with ice—two or three, however, with hot water.

In Retrospect No. 50, page 39, is a case of tetanus reported by Mr. Adams, of the London Hospital, successfully treated with ice applied to the spine.

